# Development of an Electrochemical-Based Aspartate Aminotransferase Nanoparticle Ir-C Biosensor for Screening of Liver Diseases

**DOI:** 10.3390/bios2020234

**Published:** 2012-05-29

**Authors:** Chang-Jung (Alan) Hsueh, Joanne H. Wang, Liming Dai, Chung-Chiun Liu

**Affiliations:** 1 Department of Chemical Engineering and Electronics Design Center, Case Western Reserve University, 10900 Euclid Avenue, Cleveland, OH 44106, USA; E-Mail: cxl9@case.edu; 2 Warren Alpert Medical School of Brown University, 222 Richmond Street, Providence, RI 02912, USA; E-Mail: joanne_wang@brown.edu; 3 Department of Macromolecular Science and Engineering, Case Western Reserve University, 10900 Euclid Avenue, Cleveland, OH 44106, USA; E-Mail: liming.dai@case.edu

**Keywords:** liver diseases, aspartate aminotransferase, electrochemical detection, amperometric biosensor

## Abstract

Aspartate aminotransaminase (AST) is a hepatocelluar enzyme released into the bloodstream when hepatic cells are damaged, resulting in elevated blood levels of AST. A single use, disposable biosensor prototype, composed of catalytic iridium nano-particles dispersed on carbon paste, was developed to detect enzymatically-produced H_2_O_2_ in AST-mediated reactions. This biosensor is capable of measuring AST levels in a phosphate buffer and undiluted human serum over the concentration range of 0 to 0.89 μg/mL AST concentration (corresponding to 0–250 UL^−1^ specific activity). The biosensor operates at relatively low oxidation potential (+0.3 volt (V) *versus* the printed Ag/AgCl), minimizing any potential chemical interference in human serum. The measurements of AST in human serum using the biosensor compared well with those measured by standard hospital spectrophotometric assays. This Ir-C biosensor may be useful for AST measurements in the clinical environment.

## 1. Introduction

Aspartate aminotransferase (AST) is an enzyme found in various tissues of the body, and is most notably expressed in hepatic, myocardial and skeletal muscle cells [1,2]. AST is used in conjunction with other hepatic enzymes, such as alanine aminotransferase (ALT), as a clinical biomarker of liver diseases including viral and alcoholic hepatitis, cirrhosis, and metastatic carcinoma in the liver [2,3]. Elevation of AST in serum indicates hepatocellular necrosis [3]. The elevation of AST in serum may range up to 30–40 fold of the upper normal limit (40 UL^−1^) of AST [4]. Elevation of AST concentration in serum may indicate acute myocardial infarction [5], and is also associated with patients having ischemic stroke [6,7].

**Scheme 1:**
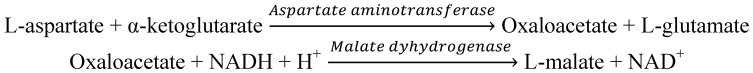
Reaction mechanism for the spectrophotometric assays of aspartate aminotransferase (AST).

The spectrophotometric technique is currently the “gold standard” in hospitals and clinical laboratories for measuring serum AST. According to the International Federation of Clinical Chemistry (IFCC) reference procedure shown in Scheme 1 [8], quantification of AST is based on the detection of an absorption change at 340 nm UV light and is associated with the formation of NADH (nicotinamide adenine dinucleotide, reduced form):

Spectrophotometry requires expensive instrumentation with complicated operational procedures. Therefore, the application of spectrophotometric detection of AST is limited and not suitable for point-of-care tracking of liver status. 

Biosensors have shown great potential in AST quantification over the past two decades. AST detection can be accomplished using a relatively small-scale, portable, inexpensive, and disposable sensor that utilizes a simple detection method. Han *et al*. modified a pyruvate oxidase (PyOx)-based biosensor on a gold electrode, incorporating oxaloacetate decarboxylase (OAC) as a coenzyme. They used a layer-by-layer technique (LBL), which exhibited the signal response through the re-oxidation of the mediator: ferrocene carboxylic acid [9]. This assay involved a complex three-step reaction mechanism in order to achieve a sensitive detection of AST within the range of 7.5–720 UL^−1^. A long incubation time (25 min) was required to allow for the generation of the oxaloacetate by-product and its reaction with the enzyme electrode. Guo *et al*. [10] developed a hydrogen peroxide (H_2_O_2_) sensor by integrating a glutamate electrode on which glutamate oxidase (GluOx) was co-immobilized with horseradish oxidase (HRP), pretreated with a redox polymer. Additionally, Ohgami *et al*. fabricated a microfluidic system by infusing a modified working electrode. This working electrode contained the identical bi-enzymes, in combination with a Y-shaped micro-flow channel for the reagents to mix through inter-diffusion [11]. These biosensor platforms showed reasonable AST detection with good linearity at a relatively rapid response time (120 s), but the reaction dependence on the assistance of coenzyme intrinsically complicated the sensor design. 

**Scheme 2:**
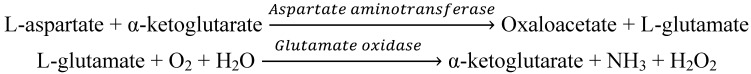
Reaction mechanism on electrochemical assays of AST by measuring enzymatically generated H_2_O_2_ from the interaction between L-glutamate and glutamate oxidase.

Simplifying the testing procedure, Song *et al.* [12], Mizutani *et al*. [13], and Ren *et al.* [14] proposed that AST activity may be effectively quantified by direct measurement of the H_2_O_2_ generated, based on a two-step reaction mechanism in Scheme 2. 

The prototype proposed by Song *et al.* did not demonstrate good linearity at low AST concentrations. This sensor for AST detection contributed to the decline in electrode stability after 120 days in storage at −4 °C [12]. Mizutani *et al.* also employed a two-step reaction mechanism, without any redox mediator in the reaction. This prototype required the use of an outer polyion membrane in order to prevent the interference of oxidizable compounds such as ascorbic (AA) and uric acids (UA) at a high applied potential (+0.8 V *versus* Ag/AgCl) for H_2_O_2_ oxidation [13]. Ren *et al.* described a Pt/Au bimetallic hierarchical electrode, using a Nafion membrane to reduce the interference of ascorbic acid and uric acid. The Nafion membrane acted as a diffusion barrier, and the sensor response was slower [14]. However, the devices described were complicated to scale up, making the production of a practical biosensor impossible.

Active carbon substrate dispersed by metallic nano-particles (NPs) possessed excellent catalytic capabilities on electroactive species such as H_2_O_2_ and NADH (nicotinamide adenine dinucleotide, reduced form) for various biosensor applications [15,16,17]. In the method proposed by Chang *et al*. [18] palladium (Pd) was blended with carbon ink as the working electrode, reducing the oxidation voltage of H_2_O_2_ to +0.4 V *versus* an Ag/AgCl reference electrode, while a Nafion coating was employed to minimize the electrode fouling. You *et al*. prepared an iridium-carbon film with nano-scaled particle distribution by co-sputtering for a glutamate sensor and measured the sensor output at the electrocatalytic potential for H_2_O_2_ reduction, thus avoiding any interference from oxidized ascorbic acid [19]. 

In our research group, a single use, disposable biosensor platform has been developed by screen-printed technology. In our development of this biosensor, a 2–5% by weight of iridium (actually iridium oxide) nano-catalyst was mixed with the active carbon powder forming the screen printable ink. The detailed fabrication of this biosensor prototype has been described elsewhere [17,19,20,21,22,23]. 

In our previous study, alanine aminotransferase (ALT) was assayed well by this iridium-dispersed carbon biosensor prototype, and our quantification of ALT in serum was in excellent agreement with the hospital spectrophotometric quantification [23]. The purpose of this current research is to develop a cost-effective, biosensor capable of quantifying serum AST using the simple two-step reaction mechanism shown in Scheme 2. The experimental quantifications of AST in human serum by this biosensor prototype correlate well with the “gold standard” spectrophotometric quantification of the hospital clinical laboratory. Therefore, this single-use, disposable biosensor provided a simple detection method for the measurement of AST in human serum.

## 2. Experimental Section

### 2.1. Materials and Reagents

L-aspartic acid, α-ketoglutararictamic acid disodium salt, L-glutamic acid, L-glutamate oxidase from Streptomyces sp. [GluOx] (E.C. 1.4.3.11), glutamic-oxalacetic transaminase from porcine heart [GOT or AST] (E.C. 2.6.1.1) were purchased from Sigma-Aldrich (St. Louis, MO, USA). Human serum was purchased from MP-Biomedicals (Solon, OH, USA). Potassium chloride, 3% hydrogen peroxide solution, sodium phosphate monobasic, and sodium phosphate dibasic heptahydrate were obtained from Fisher Scientific (Hampton, NH, USA). The Ir-C particles (5% Ir) were purchased from BASF (Somerset, NJ, USA). The additional chemicals used were of analytical grade. The buffers and other reagents made were prepared using deionized water. 

### 2.2. Fabrication Prototype of Thick-Film Screen-Printable biosensor

**Figure 1:**
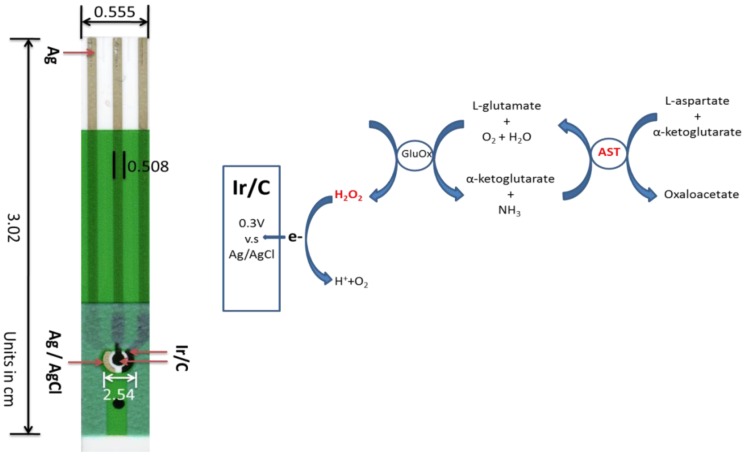
Sensor prototype and reaction mechanism used for the AST detection.

The single-use, disposable sensor platform had an overall dimension of 30 mm × 5.5 mm and was manufactured by a multi-layer approach using screen-printing technology. The detailed fabrication process had been discussed in previous publications [17,20]. Briefly, a 787 mm × 584 mm PET polymer was employed as the substrate, and silver ink was used as electrical contacts. The prepared Ir-C ink was then printed as the working and counter electrode, Ag/AgCl was printed as the reference electrode. The geometric surface area for the printed working electrode was 7.85 × 10^−3^ cm^2^. Figure 1 shows the sensor prototype which will be used for AST detection.

### 2.3. Experimental Measurement Procedure

Electrochemical measurements including voltammetric and amperometric measurements were performed using a CH Instrument 660A Electrochemical Workstation (CH Instrument, Inc., Austin, TX, USA). Measurements were at 22 ± 1 °C, unless otherwise stated. Typically, a total 200 µL testing solution was prepared with 25 mM L-aspartate, 2.5 mM α-ketoglutarate and AST reagents of variable concentration from 0 to 0.89 μg/mL (corresponding to 0**–**250 UL^−1^ in specific activity), and allowed to incubate at 37 °C to enhance the enzymatic conversion. In the testing, a 0.5 µL of glutamate oxidase (GluOx) solution of 0.05 U/µL was placed onto the biosensor prior to the testing. 0.025 U of GluOx per electrode would be produced and this amount theoretically yielded 4.5 mM L-glutamate to H_2_O_2_ per minute according to Sigma catalog, which was in excess of the testing range from 0**–**0.4 mM. All the trials were operated after the addition of 5 µL of AST testing solution with 3 minutes incubation. For the serum testing, the serum samples over the testing range of 0**–**0.89 μg/mL were prepared by spiking a variety of quantities with a known AST concentration. The identical sample preparation was applied for hospital spectrometric testing, suggesting that the serum samples tested by the Ir-C biosensor and spectrometry were compared properly. Cyclic voltammetric or amperometric measurement was then carried out to measure the AST concentration. A fresh sensor was used for each testing. Each concentration of AST was evaluated at least three times to assess the reproducibility of the biosensor. 

## 3. Results and Discussion

### 3.1. Quantification of L-Glutamate Concentration with Enzymatically Generated H_2_O_2_

**Figure 2:**
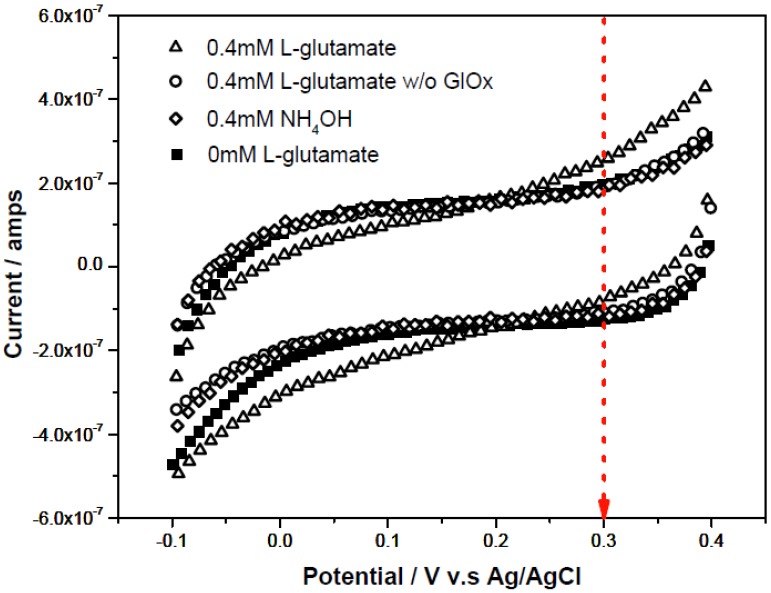
Cyclic voltammograms of background (0 mM L-glutamate) and 0.4 mM NH_4_OH, and 0.4 mM L-glutamate in a basic testing solution in the absence or presence of 0.05U/µL GluOx as testing solution (each measurement repeated 3 times using a fresh sensor).

L-aspartate and α-ketoglutarate in Scheme 2 in the presence of AST produced L-glutamate. L-glutamate reacted with glutamate oxidase producing H_2_O_2_. The L-glutamate concentration was then quantified by measuring the enzymatically-generated H_2_O_2_. In a typical run, 0.5 µL of enzyme solution (0.05 U/µL GluOx) was applied to the biosensor (Figure 1). 5 µL of L-glutamate testing solution with variable concentration 0–0.4 mM was then added to the biosensor. The reagents were prepared as 0.1 M pH 7.4 phosphate buffer solutions with 150 mM KCl supporting electrolyte. Cyclic voltammetry was performed with the scan ranges −0.1 to +0.4 V at a scan rate of 10 mV per second to identify the oxidation potential of H_2_O_2_ in the test medium. Figure 2 shows that the substrate (L-glutamate) and product (NH_4_OH) in Scheme 2 did not contribute to any oxidized currents based on the cyclic voltammograms. As shown in Figure 2 at +0.3 V *versus* the printed Ag/AgCl reference electrode, the oxidation current of H_2_O_2_ could be used to quantify the L-glutamate concentration. The low oxidation potential of H_2_O_2_ at +0.3 V minimized the interference from oxidation of other species.

**Figure 3:**
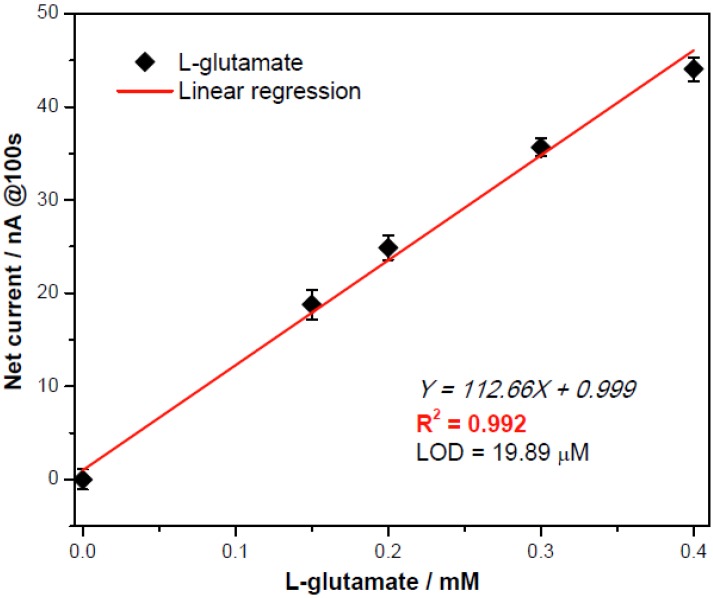
Calibration plot obtained at the 100th second for L-glutamate assessment by0.05U/µL GluOx obtained at +0.3 V *versus* the printed Ag/AgCl with repeatability demonstrated with a new sensor for each measurement (n = 3).

In Figure 3, the amperometric determination of L-glutamate concentration exhibited a good linear calibration with the resulting net currents (after subtracting from the averaged background currents) at the response time of the 100th second, quantifying L-glutamate properly and efficiently. This gave a limit of detection (LOD) = 19.89 µM of L-glutamate.

### 3.2. Determination of Aspartate Aminotransferase (AST)

#### 3.2.1. Cyclic Voltammetric Study on AST

**Figure 4:**
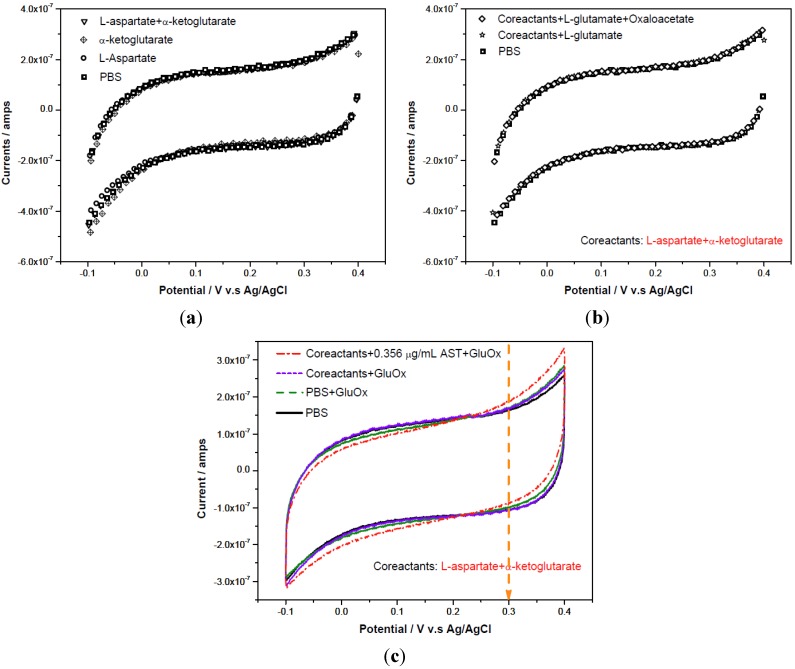
(**a**) Cyclic-voltammetric scans in the absence/presence of reactants in testing solutions: L-alanine and α-ketoglutarate; (**b**) Cyclic-voltammetric scans for different added species: reactants and products; (**c**) Cyclic-voltammetric scans for different added species in testing solutions: co-reactants in the absence/presence of 0.356 μg/mL AST and 0.5 µL of 0.05U/µL GluOx.

Cyclic voltammetry was applied to the correlated reactants (L-aspartate and α-ketoglutarate) and products (oxaloacetate and L-glutamate) involved in the AST reaction mechanism shown in Scheme 2. The cyclic voltammetric measurements were carried out in the voltage window of −0.1 V to +0.4 V *versus* the printed Ag/AgCl reference electrode with a scan rate of 10 mV per second. Figure 4(a,b) shows that there was no contribution to the current output by either chemical. Figure 4(c) shows the detection of AST by measuring the oxidation of H_2_O_2_ produced at +0.30 V *versus* the printed Ag/AgCl. 

#### 3.2.2. AST Detection with Amperometric Method

Amperometric studies of AST were carried out in phosphate buffer solution and undiluted human serum. As shown in Figure 4(c), +0.3 V *versus* the printed Ag/AgCl reference electrode was employed at this relatively low oxidation potential and any oxidation of other species was minimized. The addition of L-aspartate and α-ketoglutarate into either testing fluid would lower original pH value. Therefore, the testing medium was adjusted with 0.1 g/mL NaOH to a pH 7.4. The experimental procedures are described in Section 2.3.

**Figure 5:**
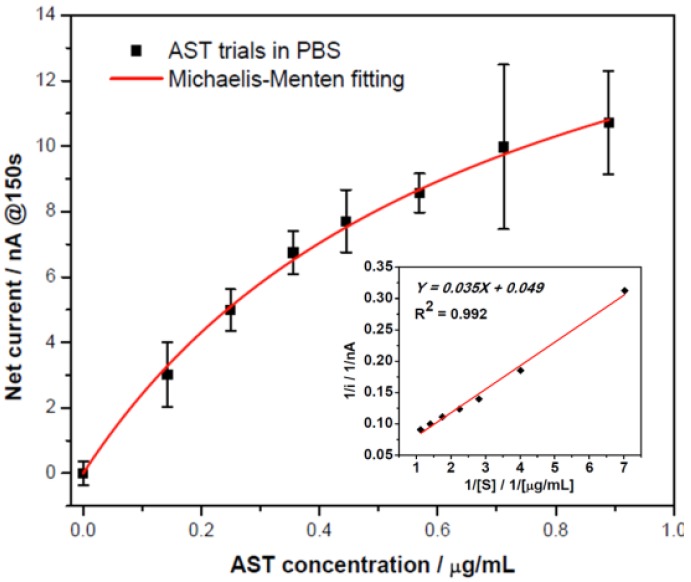
Calibration curve of ALT detection in a pH 7.4 phosphate buffer with the inset as the corresponding Lineweaver-Burk plot.

The levels of AST, ranging from 0 to 0.89 μg/mL (corresponding to AST specific activity of 0–250 UL^−1^), in phosphate buffer solution were measured. The currents at the 150th second were recorded as a function of the AST concentrations. After the subtraction of the blank (the current of 0 μg/mL AST), Figure 5(a) established the sensor current output over AST concentrations. A Michaelis-Menten relationship was obtained, and the inset in Figure 5 corresponds to the Lineweaver-Burk plot. Therefore, the apparent Michaelis-Menten constant (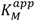 = 0.71 μg/mL) and maximum catalytic current (i_max_ = 20.4 nA) were obtained. At low AST concentrations (between 0 to 0.445 μg/mL) the calibration curve showed linearly with sensitivity of 18.5 nA/[μg/mL].

**Figure 6:**
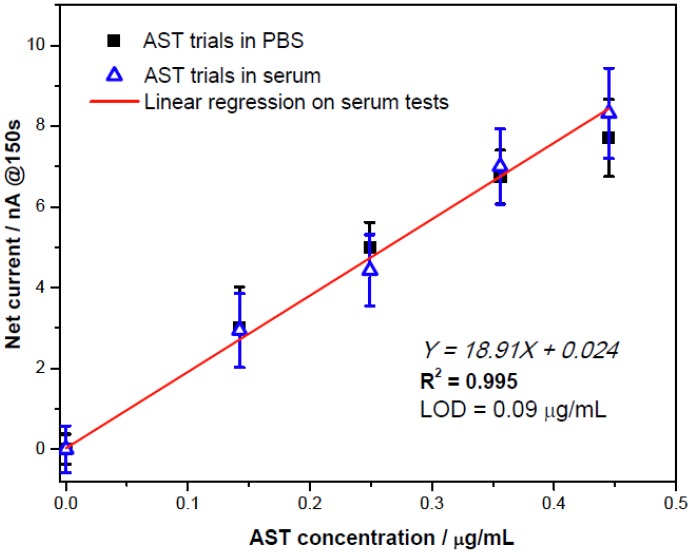
Comparable results for ALT detection in phosphate buffer and human serum testing.

**Figure 7:**
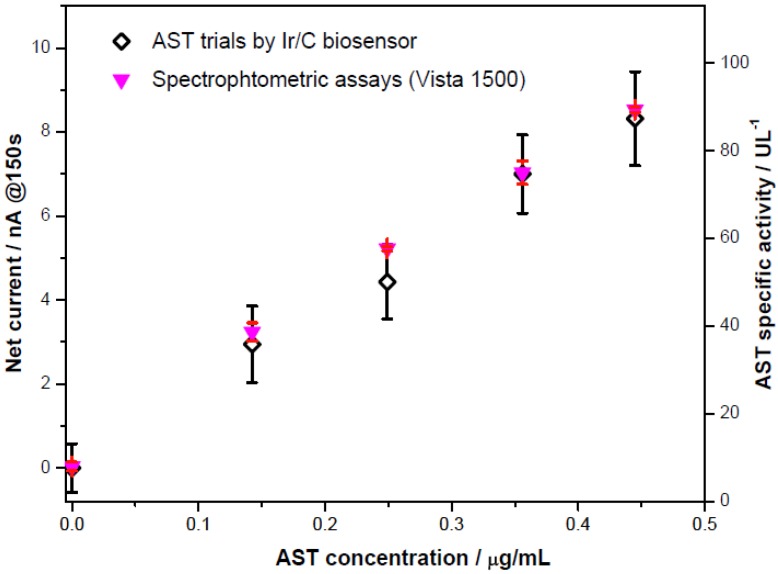
Comparison of measurements achieved by a Vista 1500 spectrophotometer to the Ir-C sensor response currents of human serum.

The quantification of AST in human serum was carried out for potential clinical applications. 100 mM potassium chloride solution was added into an undiluted serum solution as the supportive electrolyte. Figure 6 shows that the amperometric current outputs recorded at the 150th second exhibited a sensitivity, 18.91 nA/[μg/mL] (corresponding to 0.067 nA/UL^−1^), which was almost identical to that in the phosphate buffer solution. This sensitivity was higher than the experimental results in the QC serum measurement reported by Guo *et al*. [10]. Serum quantification had the limit of detection LOD = 0.09 μg/mL, corresponding to an AST specific activity of 25.3 UL^−1^. These quantifications were then compared with the assessments by Spectrophotometry Vista 1500 in the University Hospitals of Cleveland, Ohio. Figure 7 showed that the AST detection by the Ir-C biosensor and the spectrophotometric results, indicating the biosensor prototype, can be useful in detecting AST more than 3-fold of the normal upper limits (around 125 UL^−1^) in a serum sample. Therefore, the proposed Ir-C biosensor may have clinical utility in *in vitro* AST quantification in human serum samples.

## 4. Conclusions

A single-use, screen-printable Ir-C biosensor was demonstrated to be capable of AST detection using a simple two-step enzymatic reaction mechanism as shown in Scheme 2. The amperometric measurement of AST was performed by quantifying the enzymatically-produced H_2_O_2_ at an oxidation potential of +0.3 V *versus* the printed Ag/AgCl reference electrode. The biosensor’s experimental results exhibited an excellent linear relationship in both phosphate buffer and undiluted human serum over the AST concentration range of 0 to 0.445 μg/mL. Additionally, the biosensor’s assessment of AST in human serum agreed well and was compatible with the clinical spectrophotometric results obtained from the hospital.
